# Metformin and statin use associate with plasma protein *N*-glycosylation in people with type 2 diabetes

**DOI:** 10.1136/bmjdrc-2020-001230

**Published:** 2020-07-02

**Authors:** Sunny S Singh, Annemieke Naber, Viktoria Dotz, Emma Schoep, Elham Memarian, Roderick C Slieker, Petra J M Elders, Gerda Vreeker, Simone Nicolardi, Manfred Wuhrer, Eric J G Sijbrands, Aloysius G Lieverse, Leen M 't Hart, Mandy van Hoek

**Affiliations:** 1 Internal Medicine, Erasmus MC, Rotterdam, The Netherlands; 2 Internal Medicine, Maxima Medical Centre, Eindhoven, Noord-Brabant, The Netherlands; 3 Center for Proteomics and Metabolomics, LUMC, Leiden, Zuid-Holland, The Netherlands; 4 Cell and Chemical Biology, LUMC, Leiden, Zuid-Holland, The Netherlands; 5 Research Laboratory, Genos Glycoscience, Zagreb, Croatia; 6 Department of Epidemiology and Biostatistics, VUMC, Amsterdam, Noord-Holland, The Netherlands; 7 Department of General Practice and Elderly Care, Amsterdam Public Health Research Institute, Amsterdam UMC—Locatie VUMC, Amsterdam, Noord-Holland, The Netherlands

**Keywords:** glycosylated proteins, glycosylation, metformin, lipid medication

## Abstract

**Introduction:**

Recent studies revealed *N*-glycosylation signatures of type 2 diabetes, inflammation and cardiovascular risk factors. Most people with diabetes use medication to reduce cardiovascular risk. The association of these medications with the plasma *N*-glycome is largely unknown. We investigated the associations of metformin, statin, ACE inhibitor/angiotensin II receptor blocker (ARB), sulfonylurea (SU) derivatives and insulin use with the total plasma *N*-glycome in type 2 diabetes.

**Research design and methods:**

After enzymatic release from glycoproteins, *N*-glycans were measured by matrix-assisted laser desorption/ionization mass spectrometry in the DiaGene (n=1815) and Hoorn Diabetes Care System (n=1518) cohorts. Multiple linear regression was used to investigate associations with medication, adjusted for clinical characteristics. Results were meta-analyzed and corrected for multiple comparisons.

**Results:**

Metformin and statins were associated with decreased fucosylation and increased galactosylation and sialylation in glycans unrelated to immunoglobulin G. Bisection was increased within diantennary fucosylated non-sialylated glycans, but decreased within diantennary fucosylated sialylated glycans. Only few glycans were associated with ACE inhibitor/ARBs, while none associated with insulin and SU derivative use.

**Conclusions:**

We conclude that metformin and statins associate with a total plasma *N*-glycome signature in type 2 diabetes. Further studies are needed to determine the causality of these relations, and future *N*-glycomic research should consider medication a potential confounder.

**Significance of this study**

**What is already known about this subject?**

- *N*-glycosylation is a common co-translational and post-translational modification of proteins, influencing their function.
- Recent studies revealed *N*-glycosylation signatures of type 2 diabetes and cardiovascular risk factors.
- Individuals with type 2 diabetes often use medication to reduce their disease complication risk, but is it unknown how this medication affects the *N*-glycosylation profile.

**What are the new findings?**

- Metformin and statin use was associated with similar *N*-glycosylation patterns.
- These patterns were characterized by decreased fucosylation and increased galactosylation and sialylation in glycans unrelated to immunoglobulin G, increased bisection in diantennary fucosylated non-sialylated glycans, and decreased bisection in diantennary fucosylated sialylated glycans.

**How might these results change the focus of research or clinical practice?**

- The findings may point to a common biological effect in metformin and statin use. Further studies are needed to determine the causal direction of the findings and future *N*-glycomic research should consider medication as a potential confounder.

## Introduction

Type 2 diabetes is a disease with vast morbidity and mortality, mainly due to its microvascular and macrovascular complications. Many patients with type 2 diabetes use metformin, sulfonylurea (SU) derivatives, insulin, ACE inhibitors, angiotensin II receptor blockers (ARB) and statins to reduce the vascular complication risk.^1^ Some mechanisms of action of these agents are not completely understood. As the *N-*glycome is involved in virtually all (patho)physiological processes,^2^ medication use could be associated with specific total plasma *N*-glycome signatures, which might help elucidate biological pathways of these drugs. To our knowledge, the separate and simultaneous effect of metformin, SU derivatives, insulin, ACE inhibitors/ARBs and statin on the total plasma *N*-glycome has never been investigated.

Glycosylation is a common co-translational and post-translational modification of proteins, influencing their function.^3 4^ *N*-glycans affect the stability, activity and targeting of proteins, as well as cell-cell and host-pathogen interaction.^3 5 6^ These complex oligosaccharides are assembled by the concerted action of various glycosyltransferases and glycosidases, and are attached to the nitrogen (N) atom of asparagine side chains of proteins within a specific sequon.^3^ The composition of the total plasma *N*-glycome differs between individuals but remains stable in a single individual under constant physiological conditions. The total plasma *N*-glycome represents the interaction between the variants of the glycan genes and the environment. The *N*-glycome, therefore, provides key insights into the genetic, metabolic and environmental background including effects of diseases.^7^ Changes in the *N*-glycome of immunoglobulin (Ig) G and total plasma proteins have been found in aging and multiple pathophysiological conditions of different etiology, including type 2 diabetes.^8–10^

About 70% of patients with type 2 diabetes in the Netherlands use medication.^1^ Many of them use either metformin, SU derivatives, insulin, ACE inhibitors/ARB, statins or a combination of these. These medications might influence inflammation.^11 12^ Keser *et al* found an association between statins and a pro-inflammatory IgG glycomic pattern in two population-based cohorts, however, this could not be confirmed in a randomized controlled trial with rosuvastatin.^13^ Moreover, the glycomic profiles of other circulating proteins involved in the pathophysiology of diabetes, such as acute-phase proteins and apolipoproteins, have not been investigated so far. Here, we assessed for the first time the associations of the total plasma *N*-glycome with the use of metformin, SU derivatives, insulin, ACE inhibitors/ARB and statins in patients with type 2 diabetes by meta-analyzing the cross-sectional data of two large cohorts, ie, the DiaGene study^14^ and Hoorn Diabetes Care System (DCS) cohort.^15^

## Research design and methods

### Study setting and population

We used cross-sectional data from two studies in the Netherlands, a cohort of patients with type 2 diabetes from primary and secondary care, the DiaGene study and a cohort of patients with type 2 diabetes from the primary care only, the Hoorn DCS study. For both cohorts, in accordance with American Diabetes Association and WHO guidelines,^16 17^ type 2 diabetes diagnosis was defined as a fasting plasma glucose ≥7.0 mmol/L and/or a non-fasting plasma glucose level ≥11.1 mmol/L measured at least at two separate time points, treatment with oral glucose-lowering medication or insulin and/or the diagnosis by a medical specialist.

The DiaGene study has been described in more detail elsewhere.^14^ Briefly, this case-control cohort comprises 1886 patients with type 2 diabetes from all lines of care and 854 controls, from the areas of Eindhoven and Veldhoven, in the Netherlands. After data quality control, total plasma *N*-glycome data were available in 1815 cases.

The Hoorn DCS study has been described in more detail elsewhere.^15^ In short, primary care patients with type 2 diabetes from the region of West Friesland in the Netherlands visit the DCS research center annually for routine diabetes care (n=14 000). Biobanking materials, anthropometric, clinical, biochemical data and information on annual examinations for microvascular and macrovascular complications have been collected for ~6000 persons who agreed to participate in the DCS biobanks. For this particular study, we randomly chose plasma samples of 1518 subjects who donated a sample in 2008/2009. After data quality control, total plasma *N*-glycome data were available in 1518 cases.

### Patient characteristics and definitions

For both studies, clinical information on medical history, biometrics, laboratory measurements, medication use and lifestyle (ie, smoking and alcohol use) was obtained at baseline, as described.^14 15^ Mean arterial pressure (MAP) was defined as ((2×diastolic blood pressure+systolic blood pressure)/3). Cardiovascular disease (CVD) was defined as the presence of ischemic heart disease, ischemic brain disease or peripheral artery disease. Non-high-density lipoprotein (non-HDL) was calculated by subtracting HDL from total cholesterol. Creatinine was used to calculate the estimated glomerular filtration rate (eGFR) by the Modification of Diet in Renal Disease (MDRD) method.^18^ Diabetic nephropathy was defined as microalbuminuria (urine albumin/creatinine ratio (ACR) ≥2.5 for men or ≥3.5 for women) at two of three consecutive measurements, or when high microalbuminuria or macroalbuminuria was present at one measurement (ACR ≥12.5 for men and ≥17.5 for women).^14^

SU derivatives included glibenclamide, tolbutamide, gliclazide and glimepiride. Insulin included rapid-acting analogs, intermediate-acting analogs, premixed insulins and/or long-acting analogs. ACE inhibitors included captopril, enalapril, lisinopril, perindopril, ramipril and quinapril. ARBs included losartan, valsartan, irbesartan, candesartan, telmisartan andolmesartan. ACE inhibitors and ARBs were analyzed as a composite category. Statins included simvastatin, pravastatin, fluvastatin, atorvastatin and rosuvastatin. Anatomic Therapeutic Chemical codes are provided in online supplementary table 1.

10.1136/bmjdrc-2020-001230.supp1 Supplementary data

### *N*-glycome analysis and data quality control

The analysis of the total plasma *N*-glycome of the DiaGene cohort is described by Dotz *et al*,^19^ based on the workflow from Reiding *et al*,^20 21^ whereas the samples from the Hoorn DCS cohort were analyzed using the recently developed method published by Vreeker *et al*.^22^ In short, after enzymatic glycan release from plasma glycoproteins and linkage-specific sialic acid derivatization, total plasma *N*-glycome was measured by matrix-assisted laser desorption/ionization mass spectrometry, employing time-of-flight on a Bruker ultrafleXtreme instrument in DiaGene and Fourier transform ion cyclotron resonance in Hoorn DCS, using a Bruker 15T solariX XR mass spectrometer. The raw mass spectra from Hoorn DCS were calibrated in Compass DataAnalysis (Bruker Daltonics, Bremen, Germany) on an internal mass spectrum calibration list comprising 11 glycan compositions which are highly abundant in plasma. Mass spectra and glycan analytes in both cohorts were checked for quality and excluded in case of low intensity and/or interferences, as described by Dotz *et al.*^19^ Seventy-three (DiaGene) and 68 (Hoorn DCS) direct glycan compositions passed the quality control criteria, and their relative intensities were calculated by normalization to their sum, batch correction was performed and values were centered and scaled by subtracting the mean and dividing by the SD. 70 (DiaGene) and 68 (Hoorn/DCS) direct glycan compositions were further used to calculate the 45 derived traits based on their structural similarities (online supplementary table 2). Regarding the calculation of the derived traits in the DiaGene, 3 of the 73 direct traits were excluded, since it was not possible to assign them to specific structural groups.

10.1136/bmjdrc-2020-001230.supp2 Supplementary data

## Statistical analysis

To compare cohort characteristics per medication, the independent samples T-test and the Wilcoxon rank sum test were applied for continuous variables with normal and non-normal distributions, respectively. The χ^2^ test was applied for categorical variables. Normality was assumed when skewness and kurtosis were within the range of −1 and +1 (online supplementary tables 3,4).

10.1136/bmjdrc-2020-001230.supp3 Supplementary data

10.1136/bmjdrc-2020-001230.supp4 Supplementary data

The association of total plasma *N*-glycome profiles with the use of metformin, SU derivatives, insulin, ACE inhibitors/ARBs and statin was analyzed using multiple linear regression, separately for each of the medication classes. The total plasma *N*-glycome was the dependent variable and either metformin, SU derivatives, insulin, ACE inhibitors/ARB and statins were the independent variables. The basic model for each medication class included age, sex and their interaction, to reflect broad differences of users and non-users of the medication. An extended full model adjusted for specific confounders was constructed, separate for each medication or composite medication category. These full models always contained age, sex and their interaction, body mass index (BMI), HDL, non-HDL, CVD, duration of diabetes and eGFR MDRD. In the analyses of metformin, SU derivatives and insulin, we additionally adjusted for hemoglobin A1c (HbA1c); in ACE inhibitors/ARB we additionally adjusted for diabetic nephropathy and MAP and in statins for former and current smoking. Associations of *N*-glycans with type 2 diabetes were assessed with logistic regression, the following covariates were included in the model, age, sex and their interaction, BMI, HDL, non-HDL, smoking.

To address the issue of individuals using more than one of the investigated medication simultaneously, we performed additional analyses adding either metformin, insulin, SU derivatives, statin and/or the interaction of metformin and statin to the full models. Furthermore, we performed subgroup analyses for metformin, ACE inhibitors/ARB and statin in participants that used only one of these three investigated medication types. R V.3.6.0^23^ was used for analyses in the Hoorn DCS and IBM SPSS 24.0 in the DiaGene study.

The results from the linear and logistic regression on medication and case-control data, respectively, were meta-analysed using a random effects model for the two cohorts using the package ‘meta’.^24^ Correction for multiple testing was performed by the Benjamini-Hochberg procedure^25^ with a false discovery rate of <5%, with a cut-off of Q=0.05.

## Results

### Cohort characteristics

Cohort characteristics of both studies are shown in table 1, and cohort characteristics per medication class are shown in online supplementary tables 3,4. In the DiaGene study compared with Hoorn/DCS, individuals had a longer duration of type 2 diabetes (10.04 vs 7.17 years), higher HbA1c (7.03 vs 6.78 mmol/mol), higher HDL (1.71 vs 1.18 mmol/L), worse kidney function (eGFR MDRD: 77.30 vs 84.30 mL/min), a higher percentage of patients were former smokers (former smokers 56.2% vs 21.3 %) and had prevalent CVD (34.6% vs 15.5%). The percentage of use of several medications differed across the two cohorts where more individuals in the DiaGene study used SU derivatives (28.4% vs 15.5 %) and ACE inhibitor/ARB (53.3% vs 37.2 %). In contrast, more individuals in Hoorn DCS used metformin (68.3% vs 51.1 %). Percentage of insulin and statin use were comparable between the two studies.

**Table 1:** DiaGene and Hoorn DCS baseline cohort characteristics

	DiaGene (n=1815)	Hoorn DCS (n=1518)
Male sex, n (%)	977 (53.80)	854 (56.26)
Age (year)	65.21 (10.58)	64.48 (10.63)
Age of onset of diabetes (year)	54.94 (11.73)	57.29 (11.03)
Duration of diabetes (year)	10.04 (8.43)	7.17 (5.81)
BMI (kg/m^2^)	30.46 (5.45)	30.39 (5.40)
HbA1c (mmol/mol)	53.31 (11.58)	50.63 (11.33)
HbA1c (%)	7.03 (1.06)	6.78 (1.04)
MAP (mm Hg)	98.90 (10.82)	98.82 (11.31)
Total cholesterol (mmol/L)	4.29 (0.93)	4.63 (1.65)
Triglycerides (mmol/L)	1.70 (1.13)	1.82 (1.05)
HDL-cholesterol (mmol/L)	1.71 (0.32)	1.18 (0.39)
Non-HDL-cholesterol (mmol/L)	3.12 (0.90)	3.46 (1.48)
LDL-cholesterol (mmol/L)	2.45 (0.83)	2.62 (0.89)
eGFR MDRD	77.30 (22.47)	84.30 (21.73)
Smoking, n (%)		
Never	25.9	60.5
Former	56.2	21.3
Current	17.9	17.3
Cardiovascular disease (%)	34.6	15.5
Nephropathy (%)	20.2	19
Medication use (%)		
Metformin	51.1	68.3
SU derivatives	28.4	15.5
Insulin	29.6	25.1
ACE inhibitors/ARB	53.3	37.2
Statins	64.4	69.6

Unless stated otherwise, mean (±SD) are given.

ARB, angiotensin II receptor blocker; BMI, body mass index; DCS, Diabetes Care System; eGFR, estimated glomerular filtration rate; HbA1c, hemoglobin A1c; HDL, high-density lipoprotein; LDL, low-density lipoprotein; MAP, mean arterial pressure; MDRD, Modification of Diet in Renal Disease; SU, sulfonylurea.

### Medication and plasma *N*-glycome

Meta-analyzed significant associations of the total plasma *N*-glycome with medication use in the full models are summarized in table 2. Overall, there was little evidence for heterogeneity between both cohorts (data not shown). Moreover, for comparison, table 2 shows the statistics for the associations of the same glycan traits with type 2 diabetes. The complete list of outcomes for basic, full and all additional models are shown per medication category in online supplementary tables 5–9.

10.1136/bmjdrc-2020-001230.supp5 Supplementary data

10.1136/bmjdrc-2020-001230.supp6 Supplementary data

10.1136/bmjdrc-2020-001230.supp7 Supplementary data

10.1136/bmjdrc-2020-001230.supp8 Supplementary data

10.1136/bmjdrc-2020-001230.supp9 Supplementary data

**Table 2:** Meta-analyzed associations of *N*-glycan traits with medication use in the full models

N (users/non-users)		Metformin		Statin		ACE inhibitor/ARB		Type 2 diabetes vs controls	
		1740	1070	1918	804	1268	1434		
Glycan trait	Description	Beta	P value	Beta	P value	Beta	P value	Beta	P value
Complexity									
CA2	Relative abundance of A2 within complex-type glycans	−0.2314	5.24E-08	−0.1126	5.67E-02	−0.1206	3.38E-02	−0.1594	6.09E-03
CA3	Relative abundance of A3 within complex-type glycans	0.2504	1.93E-09	0.1205	1.01E-01	0.1105	5.71E-02	0.1856	1.39E-03
MHy	Ratio of high mannose-to-hybrid glycans	0.0434	3.60E-01	0.1705	3.76E-04	0.0630	7.11E-01	−0.1496	2.13E-01
Fucosylation (F)									
A2F	In A2	−0.2568	3.40E-09	−0.2447	2.36E-07	−0.1717	6.83E-02	−0.2248	5.63E-05
A2EF	In A2 with α2.6-sialylation	−0.2205	9.97E-07	−0.2611	5.45E-08	−0.1720	1.22E-02	−0.0435	6.82E-01
A2E0F	In A2 without α2.6-sialylation	−0.2044	7.26E-06	−0.1340	8.28E-03	−0.1065	6.81E-02	−0.2654	3.73E-06
A2LF	In A2 with α2.3-sialylation	−0.2370	1.98E-08	−0.2719	6.92E-09	−0.1202	1.30E-01	0.10146	2.83E-01
A2L0F	In A2 without α2.3-sialylation	−0.2411	4.38E-08	−0.2252	1.87E-06	−0.1603	6.81E-02	−0.2370	1.94E-05
A2S0F	In A2 without sialylation	−0.1378	1.53E-01	−0.0461	4.38E-01	−0.0779	1.82E-01	0.1458	1.01E-02
A3EF	In A3 with α2.6-sialylation	−0.1712	1.88E-04	−0.2524	3.69E-08	−0.0919	8.09E-02	−0.0936	1.17E-01
A3LF	In A3 with α2.3-sialylation	−0.1737	1.53E-04	−0.2449	5.89E-08	−0.0798	1.41E-01	−0.0394	5.37E-01
A3L0F	In A3 without α2.3-sialylation	−0.2183	9.52E-04	−0.3259	3.85E-06	−0.1621	1.50E-03	−0.1980	2.05E-03
A4F	In A4	−0.1768	9.97E-05	−0.2198	1.17E-06	−0.0153	7.31E-02	−0.0686	2.66E-01
Galactosylation (G)									
A2FG	Per antenna within fucosylated A2	0.1080	9.93E-02	−0.0191	9.34E-01	−0.0191	9.34E-01	0.2780	8.22E-07
A2F0G	Per antenna in non-fucosylated A2	0.2062	1.10E-02	0.1359	8.28E-03	0.1125	5.04E-01	0.2651	4.93E-06
A2FS0G	Per antenna within fucosylated non-sialylated A2	0.0260	9.79E-01	−0.0132	9.34E-01	−0.0132	9.34E-01	−0.1549	1.43E-02
A2SG	Per antenna in sialylated A2	0.2648	8.79E-10	0.1925	7.14E-05	0.1259	8.09E-02	0.2650	5.97E-07
Bisection (B)									
A2FSB	In fucosylated sialylated A2	−0.1482	4.29E-03	−0.1376	1.15E-02	−0.0700	2.08E-01	−0.1341	2.68E-01
A2FS0B	In fucosylated non-sialylated A2	0.2694	3.54E-11	0.1214	1.09E-02	0.0116	9.17E-01	0.4347	1.98E-12
A2F0S0B	In non-fucosylated non-sialylated A2	−0.0306	9.79E-01	−0.0385	9.34E-01	−0.0385	9.34E-01	0.1478	1.11E-02
Sialylation (S)									
A2GS	Per galactose within A2	0.2032	9.61E-02	0.1173	2.06E-02	0.1063	2.98E-01	0.4359	6.40E-04
A2FGS	Per galactose within fucosylated A2	0.1608	3.28E-01	0.0050	9.49E-01	0.0254	8.97E-01	0.5749	2.65E-08
A3S	Per antenna within A4	0.0867	3.93E-01	−0.0103	9.34E-01	0.0444	5.04E-01	0.1545	6.09E-03
A4S	In A4	−0.1214	4.72E-02	−0.0515	3.59E-01	−0.0692	2.08E-01	−0.2984	2.08E-07
A4FGS	Per galactose within fucosylated A4	−0.0996	1.99E-01	−0.0781	1.23E-01	−0.0756	1.82E-01	−0.2186	1.03E-04
α2.6-sialylation (E)									
A2E	Per antenna within A2	0.2128	4.48E-05	0.1122	2.51E-02	0.1240	2.08E-01	0.4888	3.03E-04
A2FGE	Per galactose within fucosylated A2	0.1060	6.29E-01	−0.0292	8.43E-01	−0.0069	9.54E-01	0.5371	1.98E-03
A3E	Per antenna within A3	−0.0291	9.79E-01	−0.0959	4.73E-02	0.0653	2.08E-01	0.4274	1.71E-11
A4E	Per antenna in A4	−0.1324	1.47E-02	−0.0187	9.34E-01	−0.0153	9.22E-01	0.3450	8.76E-09
A4FGE	Per galactose within fucosylated A4	−0.0987	1.99E-01	−0.1249	1.19E-02	−0.0047	9.54E-01	0.4231	9.36E-02
A4F0GE	Per galactose in non-fucosylated A4	−0.1560	1.57E-03	0.0050	9.70E-01	−0.0318	7.74E-01	0.2734	3.73E-06
α2.3-sialylation (L)									
A4FGL	Per galactose within fucosylated A4	0.0234	9.79E-01	0.0541	3.52E-01	−0.0271	8.63E-01	−0.4009	2.11E-03
A4F0GL	Per galactose within non-fucosylated A4	0.1086	9.93E-02	0.0075	9.49E-01	0.0125	9.17E-01	−0.3826	2.06E-10
A2F0GL	Per galactose within fucosylated A2	0.1100	9.93E-02	0.1205	1.48E-02	0.0044	9.54E-01	−0.2108	1.18E-04
A3L	Per antenna within A3	0.0884	3.15E-01	0.0575	2.80E-01	−0.0088	9.41E-01	−0.4902	5.73E-14
A4L	Per antenna within A4	0.0496	9.79E-01	−0.0149	9.34E-01	−0.0216	8.97E-01	−0.4467	3.50E-13
IgG-related									
TA2FS0	Fucosylated non-sialylated A2 in total	−0.2497	7.91E-09	−0.1471	3.19E-03	−0.1235	9.95E-02	−0.0378	3.50E-13

Regression coefficient (beta) and adjusted p values are shown per association. Blue: negative associations, red: positive associations, green: adjusted p values significant after correction according to the Benjamini-Hochberg procedure for multiple comparison. The full models always contain age, sex, the interaction thereof, BMI, HDL, non-HDL, CVD, duration of diabetes and eGFR; for metformin, SU derivatives and insulin, additional adjustment for HbA1c, for ACE inhibitors/ARB additional adjustment for diabetic nephropathy and MAP and for statins additional adjustment for smoking (ever and current) was performed. In the two outmost right columns, ‘type 2 diabetes vs controls’, the meta-analyzed associations of these glycan traits with type 2 diabetes are shown, according to model 3 from Dotz *et al*^19^ which was adjusted for the covariates age, sex, the interaction thereof, BMI, HDL, non-HDL and smoking.

ARB, angiotensin II receptor blocker; BMI, body mass index; CVD, cardiovascular disease; eGFR, estimated glomerular filtration rate; HbA1c, hemoglobin A1c; HDL, high-density lipoprotein; MAP, mean arterial pressure; MHy, mannose-to-hybrid glycans; SU, sulfonylurea.

### Metformin

The basic model of metformin use in type 2 diabetes (users n=1964, non-users n=1266) showed significant associations with several derived glycans, of which many remained significant in the full model. The strongest associations with metformin use were a decreased fucosylation in diantennary, triantennary and tetra-antennary traits (eg, A2F, A3L0F, A4F) and an increase of galactosylation in diantennary glycans, mainly in non-fucosylated and sialylated species (A2F0G, A2SG). There was a decrease in the total abundance (TA2FS0) and an increase of bisection (A2FS0B) of fucosylated, non-sialylated diantennary species. On the contrary, bisection of sialylated, fucosylated diantennary structures (A2FSB) was decreased in metformin users. Alpha2,6-sialylation of tetra-antennary glycans was negatively associated with metformin use (A4E, A4F0GE), while α2,6-sialylation per antenna in diantennary glycans was increased (A2E). Within complex glycans, the relative abundance of diantennary glycans was lower (CA2), while triantennary glycans were increased in metformin users (CA3) (table 2).

Correction for statin use did not substantially change these results. Addition of the interaction term of metformin and statin, and subgroup analysis in patients not using statins or ACE inhibitors/ARB, showed similar trends. However, some traits lost significance after adding more covariates in the equation resulting in smaller sample numbers (online supplementary table 5).

### Statins

The characteristic most strongly associated with statin use in type 2 diabetes in the full model (users n=1918, non-users n=804) was the decrease of virtually all tested fucosylated traits, especially within diantennary and triantennary structures (A2EF, A2LF, A3EF, A3L0F). Galactosylation increased in diantennary non-fucosylated (A2F0G) and in sialylated diantennary (A2SG) glycans. The relative abundance of species with bisecting GlcNAc increased within fucosylated non-sialylated glycans (A2FS0B). In contrast, bisecting GlcNAc within fucosylated sialylated glycans (A2FSB) decreased. Alpha2,6-sialylation of triantennary (A3E) and fucosylated tetra-antennary glycans (A4FGE) were negatively associated with statin use, while α2,6-sialylation per antenna in diantennary glycans increased (A2E). The higher ratio of high mannose-to-hybrid glycans (MHy) was significant in the full model (table 2).

Addition and interaction with metformin as a covariate did not substantially change the effect sizes for the derived glycan traits, however some traits lost their significance due to adding more covariates in the equation resulting in reduced power and smaller sample numbers. In the subgroup analysis of patients not using metformin or ACE inhibitors/ARB, we observed similar association patterns but only A2F, A2EF, A2LF A2L0F, A3LF, A3L0F and MM were significant due to the lower power (online supplementary table 6).

### ACE inhibitors and ARBs

In the basic model and full model (users n=1268, non-users n=1434), only three derived glycan traits were significantly associated with ACE inhibitor/ARB (CA2, A2EF, A3L0F; table 2). The observed associations are generally much weaker. Adjustment for statin and metformin use did not substantially change the results. Subgroup analysis in patients on ACE inhibitor/ARB, who did not receive metformin or statin did not render any significant outcomes probably due to the lower power (online supplementary table 7).

### Insulin and SU derivatives

The basic model of insulin use in type 2 diabetes (users n=919, non-users=2413) showed associations with several derived glycans, none of which remained significant in the full model. Adjusting for metformin use and SU derivative did not render any significant outcome (online supplementary table 8). SU derivative use (users=744, non-users=2547 in the full model) showed no significant associations with glycan traits in any of the models (online supplementary table 9).

## Discussion

We found that metformin and statins are associated with multiple structural features of plasma protein *N*-glycosylation in type 2 diabetes. Many of the traits remained significant after adjustment for potential confounders and other medication. Most of the weak associations of ACE inhibitors/ARB lost significance after adjusting for concomitant metformin and statin. SU derivatives and insulin did not show any association with the total plasma *N*-glycome despite good power.

The total plasma *N*-glycome reflects the relative abundance of glycans on proteins in the circulation. Differences in the plasma *N*-glycan profiles can, therefore, be driven by either differences in the abundance of particular *N*-glycan structures, or be caused by changes in the plasma concentration of the glycoproteins carrying these glycans. A major part of plasma glycoproteins are produced in the liver, and medication could potentially affect their production. Metformin lowers glucose most probably by reducing hepatic gluconeogenesis.^26^

Several features which were associated with metformin and statin use are similar to associations found in the meta-analyzed diabetes case-control analysis (table 2). Potentially, these disease-associated findings could have been influenced by the use of medication, since a larger portion of medication users is found in type 2 diabetes than in non-diabetic controls. Conversely, differences in the *N*-glycome of medication users and non-users could be confounded by the severity of the disease, as mildly affected patients might use less medication and may have better overall health. The *N*-glycome of more advanced type 2 diabetes differs from less severe cases and metabolic syndrome.^27^ Moreover, Keser *et al*^28^ observed different glycosylation patterns in people predisposed to type 2 diabetes, not using any of the investigated medication. Keser *et al* found that higher branching, increased sialylation and galactosylation were associated with a higher risk of type 2 diabetes and poorer regulation of blood glucose levels. Since there is no absolute measure to determine the severity of type 2 diabetes, we adjusted for HbA1c, duration of diabetes, kidney function, all risk factors for diabetes and its complications and history of CVD. This adjustment did not materially affect our observed associations.

The positive association of α2,6-sialylation of diantennary glycans (A2E) with metformin and statin is in line with earlier findings associated with diabetes.^19^ The majority of α2,6-sialylated diantennary glycans are present on acute-phase proteins, haptoglobin and IgM, and a shift in α2,6-sialylation might potentially affect their ability to bind siglec-2.^29 30^ Siglec-2 is a lectin with an important immunological function, recognizing α2,6-sialylated glycans expressed on B cells, and functioning as a molecular switch to apoptosis or activation of B cells.^31^

Metformin use was positively associated with higher branching, ie, a higher abundance of triantennary glycans (CA3). Triantennary glycans originate from acute-phase proteins, which are mainly produced in the liver during acute and chronic low-grade inflammation as is typical in type 2 diabetes. Accordingly, metformin and ACE inhibitor/ARB use were negatively associated with lower branching (CA2). Increased branching has been described in diabetes^19^ and increased risk of diabetes^32^ as well as other inflammatory diseases.^33^ The association of branching (CA3) with diabetes seems to be mediated through risk factors (eg, BMI).^19^ However, the associations with metformin remained highly significant after correcting for BMI and disease severity.

Statin use was associated with the ratio of high MHy. High mannose glycans are mostly derived from apolipoprotein B100 (apoB100), which is found on LDL and VLDL particles.^34^ Ballantyne *et al* found an elevated apoB100/non-HDL ratio in statin use.^35^ Statin use may, therefore, lower absolute apoB100 levels while increasing the apoB100/non-HDL ratio, which could explain the positive association of MHy glycans with statin use after correction for non-HDL. Another explanation can be that statins increase the glycosylation of apoB100 with mannoses.

Several glycome-medication associations were overlapping in metformin and statin use. Fucosylation of diantennary, triantennary and tetra-antennary glycans (A2F, A3F, A4F) was consistently decreased, irrespective of the presence and linkage type of sialylation. A decrease of A2F has previously been associated with type 2 diabetes itself,^19^ acute inflammation^36^ and increased C reactive protein (CRP).^37^ The majority of fucosylated diantennary glycans in plasma are thought to be derived from Igs.^34^ The total abundance of fucosylated, non-sialylated diantennary species (TA2FS0) was decreased and the bisection of these glycans (A2FS0B) was increased in metformin and statin users. These glycans are mostly derived from the Fc portion of IgG.^34^ Accordingly, our finding of increased A2FS0B in statin use is in line with elevated core-fucosylated diantennary IgG glycans with bisecting *N*-acetylglucosamine (FA2B) described by Keser *et al*,^13^ which they found in two independent population-based cohorts, in which only a small percentage had type 2 diabetes. Decreased core fucosylation of IgG strongly enhances antibody-dependent cytotoxicity, while bisection can have the opposite, however, weaker effect.^38^ Our findings might, therefore, reflect a pro-inflammatory state. A2FS0B was furthermore negatively associated with HDL and non-HDL.^19^ Therefore, the non-HDL-lowering effect of statins may explain the previously reported^19^ and here confirmed (table 2) increase of A2FS0B in type 2 diabetes. The positive association of A2FS0B with statin use in our current study remained significant after correcting for non-HDL, supporting our hypothesis that this trait is mainly driven by statin use and is likely independent from lipoprotein levels. On the contrary, bisection of sialylated, fucosylated diantennary structures (A2FSB) was lower in medication users versus non-medication users. These glycans are mostly derived from IgA, IgM and the antigen-binding portion of IgG^34^ and their biological functions are largely unknown. A2FS0G, a proxy for IgG-galactosylation, known to have a strong effect on the effector functions of IgG^38^ and to be decreased in many inflammatory diseases,^10^ was never significantly associated with our analyses, similar to the findings in statin users by Keser *et al*.^13^

Another prominent similarity in metformin and statin use was an association with increased galactosylation of non-fucosylated diantennary (A2F0G) and increased galactosylation per antenna within sialyated diantennary glycans (A2SG). Relations of enzymes controlling galactosylation (B4GALT) with hyperglycemia have been described.^39^ Moreover, A2F0G has positive associations with endogenous insulin levels and negative associations with glucose/insulin ratio in healthy subjects,^37^ suggesting an association with insulin resistance. It is difficult to translate these findings from healthy subjects to treated patients, but one could speculate that the use of the drugs we assessed here may be a reflection of worse diabetic control or severity of the disease. Although we extensively corrected for HbA1c levels, diabetes duration, the presence of CVD and nephropathy, residual effects cannot be excluded.

For both metformin and statin, pleiotropic effects have been described, consisting of mainly a beneficial effect on low-grade inflammation (CRP, interleukin (IL)6).^11^ Moreover, it is known that total plasma *N*-glycome patterns associate with markers of inflammation (CRP, IL6).^37^ Metformin and statins may have similar anti-inflammatory biological pathways in low-grade inflammation.^11^ Pharmacological interaction of metformin and statin is described,^40^ resulting in reduced levels of TNF-α compared with metformin use alone.^41^ Adding the interaction term of metformin and statins to the model did not substantially change our results, in the metformin and statin analyses. The similarity of the effects of metformin and statins possibly point to some yet unknown, shared biochemical mechanism of action or a similar influence on glycosyltransferases or glycosidases in the *N*-glycosylation machinery which warrants further detailed studies.

As for the strengths of this study, this is the first report investigating the association of total plasma *N*-glycome with metformin, statin, ACE inhibitors/ARB, SU derivatives and insulin use in type 2 diabetes. Only IgG *N*-glycosylation has previously been assessed in relation to statin use, in a non-type 2 diabetes cohort.^13^ Furthermore, we used two large independent cohorts of patients with type 2 diabetes from all lines of care. Many clinical features of these patients were available for analysis, to allow correcting for possible confounders. One potential large confounder is the severity of the disease, for which, however, a direct measure does not exist. Thus, we adjusted for clinical characteristics of more advanced stages of the disease. Despite sufficient power, we also did not find an association between insulin use and the total plasma *N*-glycome. As patients on insulin users often have a longer duration of type 2 diabetes, more complications and thus a more severe type 2 diabetes, disease severity is unlikely to represent a major confounder in our study. Finally, we used a very sensitive technique to differentiate between 70 different *N*-glycans. Using our derivatization technique, the two major sialic acid linkage types, ie, α2,3-linked and α2,6-linked sialylation, with initially the same molecular weight, could be distinguished by mass spectrometry. Despite our efforts, some limitations of our study remain, most importantly, the cross-sectional design, which limits conclusions on the causes and consequences of the link between medication and variation of the *N*-glycome. At baseline, medication use documentation and blood sample collection for glycan measurement were performed. Information on treatment duration was not available and could, therefore, not be taken into account. Furthermore, our analyses covered the use of the five major types of medication, and influences of the concomitant use of other drugs cannot be excluded. The sample size of type 2 diabetes cases not using any medication besides metformin or no medication at all was too small to perform subgroup analysis. Finally, both cohorts were mainly of Caucasian descent. As ethnicity may influence glycan associations,^42^ we cannot generalize our findings on other ethnic groups.

In conclusion, a clear relationship between the total plasma *N*-glycome and the use of either metformin and statins was found. This is important to consider in future *N*-glycomic research, as medication use could be a confounder. Besides, a striking similarity of the *N*-glycome in statin and metformin use was seen. This could reveal a shared, yet unknown, mechanism of action, for example, on the glycosylation machinery. Knowledge of the influences of medication on the *N*-glycome and knowledge about the role of plasma protein *N*-glycosylation in pathophysiology could reveal new leads for disease, prevention, novel medication and treatment strategies.
